# Association of serum PCSK9 levels with platelet function in patients with acute coronary syndromes

**DOI:** 10.1097/MD.0000000000033026

**Published:** 2023-04-14

**Authors:** Wenting Wang, Ronghua Luo, Dean Pei, Qi Huang, Xinyao Jin, Yuanhong Wu, Mingbin Xie, Shisheng Wang, Xiangqian Sui, Bin Shen

**Affiliations:** a Department of Cardiology, Affiliated Hangzhou Chest Hospital, Zhejiang University School of Medicine, Hangzhou, China.

**Keywords:** acute coronary syndrome, oxidized low-density lipoprotein, platelet reactivity, proprotein convertase subtilisin/kexin type 9

## Abstract

Proprotein convertase subtilisin/kexin type 9 (PCSK9) regulates low-density lipoprotein (LDL) homeostasis and plays a key role in acute coronary syndrome (ACS). The cardioprotective effect of PCSK9 inhibition extends beyond LDL cholesterol reduction, involving regulation of platelet function by not yet unraveled mechanisms. Oxidized-LDL (ox-LDL) is increased during ACS and induces platelet activation via binding to platelet surface. We will evaluate serum PCSK9 and its correlation with platelet reactivity and platelet-ox-LDL binding in Chinese ACS patients.

**Method and design:**

In this pilot cross-sectional study, we will enroll 115 Chinese participants aged 30 to 75 years with ACS. Blood sample will be obtained after the first maintenance dose of aspirin and clopidogrel during morning time. Serum PCSK9 will be measured by an enzyme-linked immunoadsorbent assay. Platelet reactivity will be assessed by; Platelet activation (P-selectin and GPIIbIIIa expression using flow cytometry) and; Platelet aggregation using light transmission aggregometry in response to various stimuli. On-treatment platelet reactivity is measured by adenosine diphosphate-induced platelet aggregation. Binding of ox-LDL to platelet will be evaluated by flow cytometry. Spearman correlations will be used to determine association of serum PCSK9 with platelet functional parameters and platelet-ox-LDL binding. Additionally, continuous PCSK9 levels will be categorized into tertiles of equal size to investigate its association with on-treatment platelet reactivity.

**Discussion:**

This study will reveal possible relationship between serum PCSK9 and platelet reactivity in the setting of ACS which may shed light on therapeutic potential in platelet inhibition by targeting PCSK9. The study will also explore the association of serum PCSK9 and platelet-ox-LDL binding, an important mechanism for platelet-LDL interplay, to provide mechanistic insight into PCSK9-mediated regulation of platelet reactivity.

## 1. Introduction

Platelet activation and aggregation play a central role in arterial thrombosis and in the pathophysiology of acute coronary syndrome (ACS).^[1]^ Whereas despite the use of aspirin and a P2Y_12_ antagonist, a standard antiplatelet regimen in ACS, recurrent thrombotic events still occur in some patients, which is related to insufficient inhibition of platelet reactivity.^[2,3]^ The mechanisms that underlie the residual platelet hyper-reactivity in ACS is multifaceted where the involvement of low-density lipoprotein (LDL) is crucial.^[4]^

Platelets are continuously exposed to circulating LDLs in the blood stream. Furthermore, platelets are also in close contact with oxidized LDL (ox-LDL) released from the ruptured plague in the setting of ACS.^[4]^ Mounting experimental evidence suggests that both native and ox-LDLs substantially affect platelet reactivity.^[5,6]^ By binding to scavenger receptors (SRs), LDLs and ox-LDLs alter platelet signal transduction cascades, resulting in platelet hyper-sensitivity to the activating stimuli.^[7]^ More importantly, binding of ox-LDL induces platelet activation followed by shape change and aggregation, ultimately contributing to thrombus formation after plague rupture.^[8]^ In keeping with the preclinical data, Stellos et al^[9]^ reported increased platelet-ox-LDL binding in patients with ACS versus stable coronary artery disease. Therefore, the lipoprotein-platelet interplay provides a basis for the concept that regulation of ox-LDL-mediated platelet hyper-reactivity may translate into novel antiplatelet strategy for ACS patients.

Proprotein convertase subtilisin/kexin type 9 (PCSK9) fosters the degradation of LDL receptors, suppresses LDL clearance and subsequently raises circulating LDL and ox-LDL levels.^[10]^ A clinical trials has demonstrated that PCSK9 inhibition efficiently reduces recurrent cardiovascular events in ACS patients,^[11]^ which is considered largely LDL-dependent. Indeed, preliminary data also indicates other atherogenic mechanisms mediated by PCSK9 that are independent from lipid metabolism, several of which may affect platelet reactivity and thrombogenesis.^[12]^ In PCSK9^-/-^ mice, plasma level of soluble P selectin, a platelet activation biomarker,^[13]^ was significantly lower than that in control mice.^[14]^ This finding was supported by another study showing reduced FeCl_3_ injury-induced carotid artery thrombosis and platelet P selectin expression in PCSK9^-/-^ mice versus control.^[15]^ Whereas mechanistical data regarding PCSK9-mediated regulation of platelet reactivity is so far limited.

ox-LDL binds to platelet via lectin-like oxidized low-density lipoprotein-1 (LOX-1), a class E SR,^[16]^ and this interplay contributes to ox-LDL-mediated platelet hyperactivity and facilitates inflammation-driven atherothrombosis.^[17]^ Interestingly, upregulation of PCSK9 potently stimulates LOX-1 expression and ox-LDL uptake in macrophages in an inflammatory milieu.^[18]^ The above-mentioned clues support a hypothesis that PCSK9 may also be involved in modulating ox-LDL-platelet interplay, which may be associated with PCSK9-mediated regulation of platelet reactivity. This cross-sectional study will demonstrate serum PCSK9 level and its relationship with platelet reactivity and platelet-ox-LDL binding in Chinese ACS patients to address these knowledge gaps. We present the protocol in accordance with the Standard Protocol Items: Recommendations for Interventional Trials reporting checklist.

## 2. Methods/ design of the study

### 2.1. Study design and setting

The pilot study is designed as a single center cross-sectional trial, which will be conducted at the Center of Chest Pain of Affiliated Hangzhou Chest Hospital, Zhejiang University School of Medicine (Zhejiang, China). Ethical approval has been granted by the Institutional Ethics Committee of Affiliated Hangzhou Chest Hospital. This study has been registered in the Chinese Clinical Trials Registry (registration number: ChiCTR2100052578 at http://www.chictr.org.cn/). This study will follow the Strengthening the Reporting of Observational Studies in Epidemiology guidelines. We present the protocol in accordance with the Standard Protocol Items: Recommendations for Interventional Trials reporting checklist.

### 2.2. Study population and recruitment procedures

**Figure 1.**
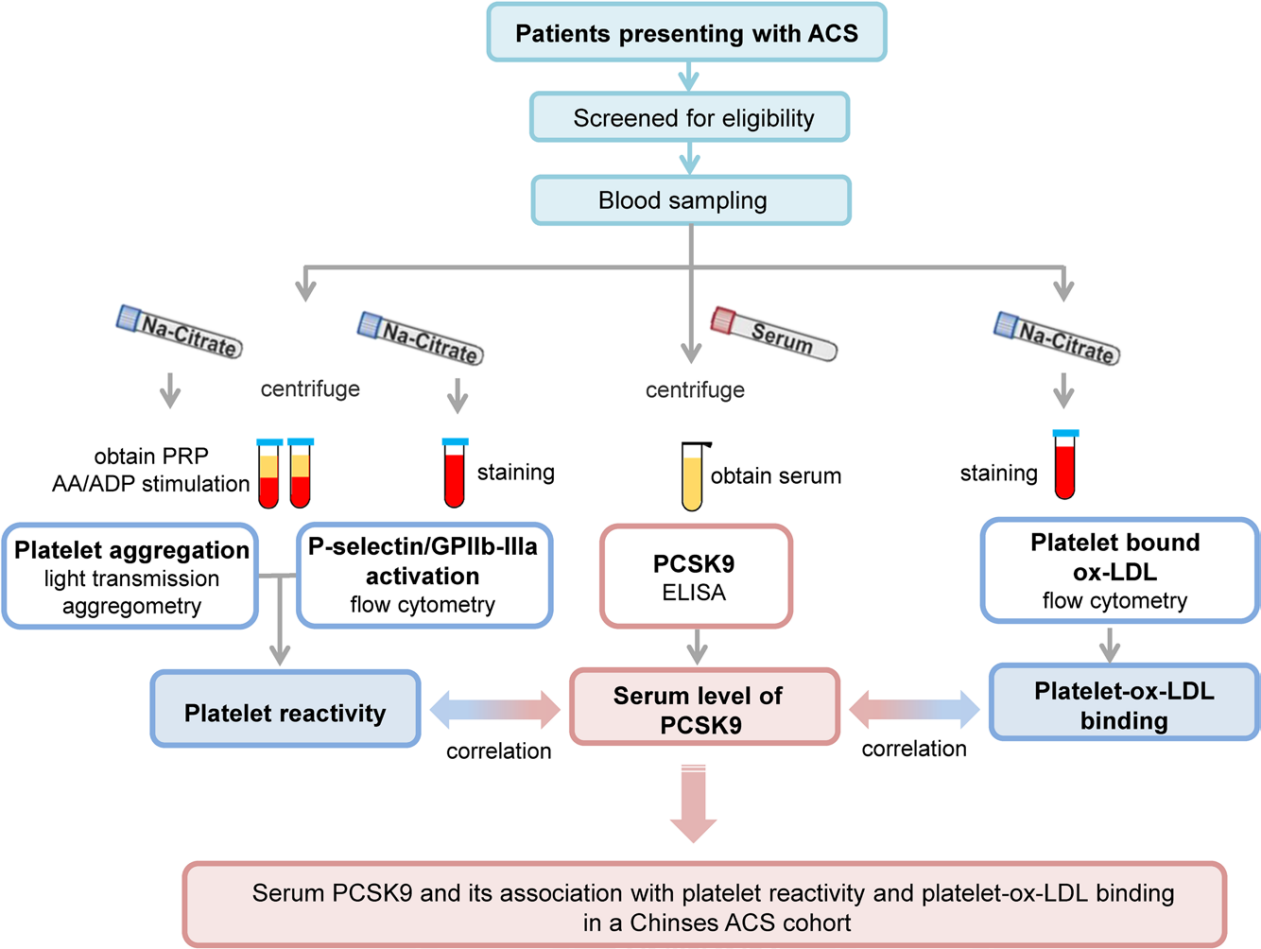
Flowchart of the study.

Patients aged 30 to 75 years presenting with ACS (unstable angina, ST-elevation or non-ST-elevation myocardial infarction) will be screened for eligibility. Voluntary patients will be enrolled after written informed consent is obtained. Immediately after the administration of loading dose of aspirin (300 mg) and clopidogrel (300 mg), blood sample will be harvested for further analysis of serum PCSK9, platelet reactivity and ox-LDL-platelet binding. To be more specifically, blood sampling will be performed before catheter procedures. Patients will be excluded with coronary stenosis < 50% as ascertained by coronary angiography. The inclusion and exclusion criteria are given in Table 1. The flowchart of the study is shown in Figure 1.

**Table 1:** Inclusion and exclusion criteria.

Inclusion	Exclusion
• Patients aged 35–75 yr	• coronary stenosis < 50% as ascertained by angiography
• Presenting with ACS, including unstable angina, ST-elevation and non-ST-elevation myocardial infarction	• uncontrolled blood pressure, heart failure pulmonary dysfunction
	• severe hepatic, renal, hematologic, mental disorders, terminal illness (carcinoma, etc)
	• pregnancy or breastfeeding
	• reluctance to sign an informed consent form

ACS = acute coronary syndrome.

### 2.3. Measurements

#### 1.2.3. Serum level of PCSK9.

The blood samples obtained from patients will be taken into tubes and stored at −4°C. The samples will be at 300 g for 10 minutes to obtain supernatant and then immediately stored at 80°C until use. Serum levels of PCSK9 will be measured by a commercial enzyme-linked centrifuged immunoadsorbent assay according to manufacturer’s instructions.

#### 2.2.3. Platelet activation biomarkers.

Platelet activation will be assessed by flow cytometry as previously described.^[19]^ Platelet-bound P selectin and GPIIb-IIIa activation, biomarkers of platelet activation, will be analyzed at rest or stimulated with adenosine diphosphate (ADP).^[20]^ Briefly, whole blood will be collected in evacuated vacuum tubes containing 3.2% trisodium citrate at room temperature. 5 µL of citrated blood will be diluted with 100 µL of phosphate-buffered solution with or without stimulation by ADP (35µmol/L). Anti-CD61 will serve as control antibody to identify platelets from the whole blood. Monoclonal antihuman antibodies will be used to measure expression of P selectin (anti-CD62p) and activated form of GPIIb-IIIa (anti-PAC-1). Unspecific binding will be excluded by relevant IgG control antibodies. After staining, the cells will be fixed with 0.25% polyformaldehyde and stored at 4°C until analysis. Specific monoclonal antibody binding will be determined as mean fluorescence intensity.

#### 3.2.3. Platelet aggregation assay.

Platelet aggregation will be analyzed using light transmission aggregometry according to standard protocols.^[21,22,23]^ Sodium citrate anticoagulated whole blood will be handled within 1 hour. Samples will be centrifugated at 260g for 10 minutes to obtain platelet rich plasma (PRP) and additionally at 760g for 10 minutes to obtain platelet-poor plasma. Platelet concentration in PRP will be adjusted to 2 × 10^8^/mL by dilution with homologous platelet-poor plasma. After stimulation of ADP (10µmol/L) or (arachidonic acid, 0.5 mmol/L), adjusted PRP will be utilized for the assay and light transmission will be monitored by an aggregometer over 6 minutes. Aggregation is quantified with 100% aggregation corresponding to 100% light transmission and maximum platelet aggregation rate (MPAR) will be used for statistical analysis. ADP- and arachidonic acid -induced platelet aggregation will be recorded as MPAR_ADP_ and MPAR_AA_, respectively. In addition, we define high on treatment platelet reactivity (HTPR) as MPAR_ADP_ >59%.^[24]^

#### 4.2.3. Binding of ox-LDL on circulating platelets.

Whole blood will be studied for platelet bound ox-LDL by flow cytometric as previously reported.^[9]^ In brief, 5 µL of sodium citrate anticoagulated blood will be suspended in100 µL of phosphate-buffered solution. Platelets in the whole blood will be identified with anti-CD61. Then samples will be incubated with a polyclonal antihuman antibody (anti-Cu^2+^-ox-LDL) to detect ox-LDL on the surface of platelets and unspecific binding will be excluded by a relevant IgG control antibody. After staining, the cells will be fixed with 0.25% polyformaldehyde and stored at 4°C until analysis with a flow cytometer.

### 2.4. Data management

To ensure the confidentiality of information, every participant will be assigned with a unique identifier linking to personal data, which is accessible by a trained research assistant. The completeness and plausibility of data from all assessments will be ensured by the responsible investigators for quality control. All study investigators will comply with the sponsor audit requirements. During all stages of the study, auditing can be carried out by independent individuals appointed by the sponsor to ensure the quality of the study and the validity of the results.

### 2.5. Statistical considerations

#### 1.2.5. Sample size.

This is a pilot study that examines the possible relationship of PCSK9 expression and its association with platelet reactivity and platelet-ox-LDL binding. A sample size between 24 and 50 participants has been recommended for a pilot study.^[25]^ In addition, the appropriate sample size of a pilot study should be calculated in relation to other similar studies.^[26]^ According to previous research,^[27]^ with the consideration of estimated attrition rate of 20%, a total of 115 patients will be recruited in the present study.

#### 2.2.5. Statistical analysis.

Categorical variables will be reported as counts (percentage). Distribution of continuous variables will be assessed by the Kolmogorov-Smirnov test. Normally-distributed continuous variables will be expressed as mean ± SD. Non-normally distributed continuous variables will be expressed as median and interquartile range (IQR). Comparisons between the groups will be performed by Pearson chi-squared test, analysis of variance or Kruskal-Wallis test, as appropriate. The association of PCSK9 levels with platelet reactivity and ox-LDL-platelet binding will be evaluated by Spearman rank test. For further analysis, we will divide the cohort into tertiles of PCSK9 values. univariate and multivariate logistic regression will be used to analyze the association between PCSK9 levels with on-treatment platelet reactivity. All tests will be 2-sided with an alpha level of 0.05. Analysis will be performed using SPSS version 20.0 (SPSS Inc., Chicago, Illinois).

## 3. Discussion

Patients who had a recent ACS remain at high risk of recurrent ischemic events despite the current evidence-based treatments, including dual antiplatelet therapy and lipid-lowering agents.^[28]^ Circulating levels of PCSK9 dynamically increase during ACS as a consequence of cardiac ischemia.^[29]^ A clinical study has revealed that PCSK9 inhibition may confer benefits during ACS through LDL-dependent mechanism.^[11]^ Intriguingly, beyond its effects on LDL metabolism, preliminary results suggest PCSK9 as a positive modulator of platelet activation in in vitro and in vivo models.^[15,30,31]^ On the basis of prior preclinical observations, we will demonstrate possible relationship between circulating PCSK9 and platelet reactivity using multiple platelet function assays in a Chinses ACS cohort. To the best of our knowledge, 1 study including Austrian ACS patients reported an association between increased serum PCSK9 levels and platelet aggregation in response to a single agonist ADP.^[27]^ It should be noted that a single measurement of platelet reactivity may lead to inaccurate evaluation of the in vivo status of circulating platelets.^[32]^ Here in our study, more detailed analysis of platelet reactivity will be undertaken by quantification of platelet activation markers and aggregation induced by various agonists.

In order to minimize confounding effects on platelet reactivity, several strengths in the methodology should be noted. For 1, to exclude any influence of antiplatelet drug effects on platelet status, only patients receiving dual antiplatelet therapy with aspirin and clopidogrel will be recruited. More specifically, blooding sampling will be taken immediately after the loading dose of aspirin (300 mg) and clopidogrel (300 mg) and before coronary angiography or percutaneous coronary intervention (PCI), because catheter procedure itself is traumatic to platelets and testing within 96 hours post-PCI seems highly unpredictable regarding ex vivo platelet reactivity.^[32,33]^ Further, experimental data revealed remarkable increase in PCSK levels at 12 to 96 hours after cardiac ischemia.^[34,35]^ In this regard, the optimal timing for testing platelet reactivity, along with its association with PCSK concentration should be prior to PCI procedures.

Preclinical studies have demonstrated that PCSK9 directly enhances platelet activation and in vivo thrombosis.^[36,30]^ Accordingly, Cammisotto et al^[37]^ found reduced platelet activation in patients with familial hypercholesterolemia after PCSK9 inhibitors administration, which was mechanistically linked to modulation of ox-LDL pathways. Indeed, during the inflammatory and oxidative processes of plaque rupture, the prothrombotic ox-LDL is generated from the modification of LDL by phospholipid oxidation.^[38]^ These observations prompt the hypothesis that PCSK9 may potentiate platelet reactivity via regulating ox-LDL-platelet interplay in the setting of ACS. Results from in vivo models revealed that ox-LDL, as a high-affinity ligand for platelet SR (e.g. LOX-1, CD36), facilitates platelet activation via binding to platelet surface.^[39]^ Interestingly, PCSK9 increased the expression of LOX-1 and ox-LDL uptake in macrophages in an inflammatory milieu.^[18]^ Whether PCSK9 is involved in the regulation of ox-LDL-platelet binding has never been evaluated in clinical settings. To this end, the association of serum PCSK9 and platelet-ox-LDL binding, an important mechanism for platelet-ox-LDL interplay, will also be explored in ACS patients.

Several limitations should be considered in this study. As this is by far the first analysis addressing the association of circulating PCSK9 with platelet reactivity and with platelet-ox-LDL binding in ACS Chinese cohort, we aim to recruit patients from a single center to achieve preliminary results. Given the limited sample size the intrinsic shortcomings of the cross-sectional design, future large prospective studies are warranted to confirm our findings. We notice that there are mounting evidence showing the direct effect of PCSK9 on platelet function in experimental conditions.^[18,30]^ In order to translate the observations into clinical settings, we emphasize to investigate the serum PCSK9 and its correlation with platelet reactivity using multiple assays, with additional goal to provide mechanistic insight from the perspective of platelet-ox-LDL interplay. If positive results are achieved, future studies are needed to unravel pathways downstream of ox-LDL-platelet binding to underlie the mechanism of PCSK9-mediated platelet activation.

## 4. Conclusions

Several preclinical clues have suggested that beyond the role in LDL metabolism, PCSK may be involved in regulating of platelet function. To address the knowledge gaps, we aim to evaluate circulating PCSK9 levels and unravel its association with platelet reactivity in a Chinese ACS cohort. If positive results are obtained, the observations would reinforce the concept of PCSK9-mediated modulation of platelet activation in a clinical setting. Moreover, we will reveal possible relationship between PCSK9 level and ox-LDL-platelet binding, an important mechanism underlying ox-LDL-induced platelet hyperactivity, to provide mechanistic insight into PCSK9-mediated platelet activation.

## Acknowledgements

The authors are sincerely thankful for expert technical assistance of professor Dazhuo Shi from Cardiovascular Center, Xiyuan Hospital, China Academy of Chinese Medical Sciences.

## Author contributions

**Methodology:** Ronghua Luo, Dean Pei, Qi Huang, Xinyao Jin, Bin Shen.

**Validation:** Shisheng Wang, Xiangqian Sui.

**Writing – original draft:** Wenting Wang.

**Writing – review & editing:** Wenting Wang, Yuanhong Wu, Mingbin Xie.
